# Influence of the Imagination of the Mother upon the Fœtus

**Published:** 1852-06

**Authors:** L. Slusser

**Affiliations:** Canal Fulton, Ohio


### *Influence of the Imagination of the Mother upon the Fœtus.*

By L. Slusser, M. D., of Canal Fulton, Ohio.

Whether the fœtus in utero can be affected in its development by extraneous causes, operating through the imagination of the mother; or, at what period of utero-gestation an arrest or perversion of development can take place, if at all; or the precise time at which those influences cease, admitting that they may be produced—are subjects upon which the profession are divided. Without designing to take either side of a question so obscure, and yet so speculative, I shall detail a case in point, allowing the reader to form his own conclusions.

Mrs. R——, aged about 34, a lady of more than ordinary strength of mind, and of cultivated intellect, was married in March, 1850. She is of sanguine temperament, and has always been regular in her menstrual function. About four months after marriage, she became pregnant; but had an abortion in the second month, excited, as she supposed, from fatigue in a long walk. Her convalescence was somewhat tedious, as she suffered considerably from uterine hemorrhage.

Her catamenia restored, continued uninterrupted until June, 1851, when they were again suppressed. The ordinary indications following, she suspected herself again pregnant, in which suspicion she was in due time confirmed by unmistakable signs. Gestation progressed without the occurrence of anything worthy of note, to the sixth month; up to that period she enjoyed unwonted health. Appetite good—bowels regular—spirits buoyant—sleep refreshing—in brief, she was entirely free from those tormenting sympathetic troubles, no less common than harassing to the pregnant female. Desirous of offspring, she looked forward with bright hopes and fond anticipations to her expected confinement.

About this time, a cub bear, brought from California, was purchased by a citizen of the town and kept in an out building of a lot adjoining her residence. The animal, unused to close confinement, kept up a strange and unearthly noise, much resembling that made by a lunatic brother of Mrs. R. who had been under her special charge for several years previous, but now an inmate of an asylum. The peculiarly distressing noise made by the animal, occasioned, as Mrs. R. supposed, by want of food, distressed her exceedingly. She had frequent opportunities of seeing the animal, which always increased her trepidation of mind, which was naturally sensitive. Every effort to control her feelings—to fortify her mind, and resist this influence, was unavailing. The bear seemed ever present in her mind. This constant excitement soon began to sensibly affect her health. Her rest became broken—appetite impaired—bowels costive; in short the whole train of sympathetic disorders frequent in her condition, were unusually aggravated. Medication afforded but temporary relief. Efforts were made to have the bear removed, but they were unavailing.

On Saturday morning, Feb. 28th ult., while yet in bed, she was awakened from sleep by a discharge of water—the membranes having ruptured spontaneously, and without pain. She immediately took a portion of oil, which operated in a few hours. During the day she had a free discharge of water, especially at every movement of the body, but she experienced no pain. In the evening I was called in—made an examination, and found the os uteri slightly dilated, but tense—the head presenting. About midnight she was delivered of a female child, much emaciated, and in a state of asphyxia. By means of external stimuli, and the substitution of artificial respiration, the child was resuscitated, when I tied the cord, and separated it from the placenta which followed soon after.

The child lived but about twenty hours. The following is a report of an examination made of it by Dr. Dorland and myself, previous to its interment.

The feet and ankles resemble talipes valgus. The soles of the feet flat, and the heels projecting beyond the usual length. The thighs flexed upon the abdomen, and the flexor muscles so contracted as not to admit of full extension, without great stretch of the tissues involved. The same was the case with the upper extremities. The arms could not be raised above a right angle with the body. The wrists turned out—the fingers over-lapped each other, and their flexor tendons were contracted. The nails were elevated in the centre, and quite pointed, much resembling claws. The arms, legs and back were covered with a fine hair from three to six lines in length, of a dark brown color, and so abundant as to strike all who beheld it with wonder and astonishment. The skin along the limbs and back was remarkably rough. In other respects, the appearance of the child was natural, though we did not examine the internal organs. The facial and cranial developments were perfect. The child had nursed, and had a motion of the bowels previous to its death, but no discharge of urine.

The physiological question involved in this case is, could the fœtus, at the period of advance indicated, (not less than six, nor more than seven months,) be susceptible of an alteration from a normal condition, presupposing that up to that period there was a perfect development? As the muscular and dermoid tissues are the last formed, could their development have been arrested from extraneous causes operating through the mind of the mother?

